# Medication supply to residential aged care facilities in Western Australia using a centralized medication chart to replace prescriptions

**DOI:** 10.1186/1471-2318-12-25

**Published:** 2012-07-11

**Authors:** Kreshnik Hoti, Jeffery Hughes, Bruce Sunderland

**Affiliations:** 1 Curtin Health and Innovation Research Institute, School of Pharmacy, Curtin University, GPO Box U1987, Perth, WA, Australia, 6845

**Keywords:** Aged care, Nursing homes, Elderly, Medication chart, Medication supply, Prescriptions

## Abstract

**Background:**

Current model of medication supply to R (RACFs) in Australia is dependent on paper-based prescriptions. This study is aimed at assessing the use of a centralized medication chart as a prescription-less model for supplying medications to RACFs.

**Methods:**

Two separate focus groups were conducted with general practitioners (GPs) and pharmacists, and another three with registered nurses (RNs) and carers combined. All focus group participants were working with RACFs. Audio-recorded data were compared with field notes, transcribed and imported into NVivo® where it was thematically analyzed.

**Results:**

A prescription-less medication chart model was supported and it appeared to potentially improve medication supply to RACF residents. Centralization of medication supply, clarification of medication orders and responding in real-time to therapy changes made by GPs were reasons for supporting the medication chart model. Pharmacists preferred an electronic version of this model. All health professionals cautioned against the need for GPs regularly reviewing the medication chart and proposed a time interval of four to six months for this review to occur. Therapy changes during weekends appeared a potential difficulty for RNs and carers whereas pharmacists cautioned about legible writing and claiming of medications dispensed according to a paper-based model. GPs cautioned on the need to monitor the amount of medications dispensed by the pharmacy.

**Conclusion:**

The current use of paper prescriptions in nursing homes was identified as burdensome. A prescription-less medication chart model was suggested to potentially improve medication supply to RACF residents. An electronic version of this model could address main potential difficulties raised.

## Background

A significant number of Australia’s elderly population are currently cared for in Residential Aged Care Facilities (RACFs) and represent a population group with a high prevalence of diseases and co-morbidities [1]. Medication supply in Australian RACFs is currently negotiated between a community pharmacy and the RACF. The pharmacy supplies medications based on general practitioner (GP) paper prescription forms which are also reproduced by prescribers on individual residents’ medication charts. This gives rise to a requirement for duplicated entries. In order to ensure quality use of medicines, the use of medication charts to record administered medicines is one of the recommendations made by the Australian Pharmaceutical Advisory Committee [2]. In Australian RACFs, medications are primarily prescribed by residents’ GPs. In addition some are prescribed by specialists, locums and hospital doctors. Registered Nurses (RN) are allowed to use their clinical judgment and assessment to initiate ‘Pharmacy Only’ and ‘Pharmacist Only’ medications for RACF residents. Dentists, optometrists and registered RN practitioners are also able to prescribe medicines in Australia [3].

Pharmacists are mainly reimbursed for dispensing and supplying medicines to RACFs through the Pharmaceutical Benefits Scheme (PBS). This does not apply for private prescriptions (i.e. medications not subsidized by the PBS), which are charged to patients. For reimbursement by the PBS, pharmacists currently need a paper prescription form for every medication dispensed, in accordance with the orders on the resident’s medication chart. The pharmacy then supplies medications to RACF residents, usually using Dose Administration Aids (DAA) or original packs depending on residents’ ability to administer medications or by arrangement with the RACF [1,4]. There may be situations where residents’ original prescriptions are not available for dispensing or repeat prescriptions run out. In these cases pharmacists are able to initiate or continue medication supply based on an ‘owing prescription’ system but the prescription must be received within seven days [5]. The ‘owing prescription’ system presents a number of important issues, notably considerable pharmacist time is consumed in following up prescriptions from GPs and delays in reimbursement whilst waiting for the prescription to be received from the GP.[5,6] Furthermore, in some cases GPs may then decide to discontinue the therapy hence leaving pharmacists without a prescription [6]. Delays in receiving prescriptions often renders the ‘owing prescription’ system in breach of the statutory requirements. Pharmacists can also supply medications based on the ‘emergency supply’ system. However, this system is associated with an increased cost burden for pharmacists due to the breaking of original packs of medicines and it also requires a high level of clerical organization for the health professionals involved [6]. The Healthcare Management Advisors (HMA) found that under the present system prescribers currently perceive the requirement of ordering medications on the medication chart as well as issuing paper prescriptions as a time consuming duplication of tasks [5].

The use of a centralized medication chart model was proposed under the Fifth Community Pharmacy Agreement [7]. This model was also supported by stakeholders in the HMA review who identified resources needed to implement this model [5]. According to this model, there would be no need for paper prescriptions and the medication chart would be the central legal document by which medication supply to RACFs would take place. A medication supply system which is based on the medication chart model was due to be implemented in Australia from 1^st^ of July 2012 and the development of a national residential medication chart is under way [8,9]. There is currently a lack of literature considering the use of medication chart models to supply medications to RACFs. The aim of this study is to explore the use of a medication chart model in RACFs.

## Methods

This study was approved by the Human Research Ethics Committee of Curtin University.

The data were collected using focus groups. This qualitative method was chosen as it enabled exploratory work to be carried out in order to assess the views of study participants. Group discussion generated through interaction of study participants contribute to a more detailed, cost-effective and timely exploration of different perspectives [10]. Participants in focus groups were currently working with at least one RACF. Focus groups consisted of GPs, pharmacists and RNs/carers. Each focus group was homogenous in terms of professions. Homogenous groups were organized since they capitalize on common experiences [11]. Two focus groups were organized with GPs and two with pharmacists. To ensure a wide representation of ideas and saturation of themes, three focus group discussions with RNs and carers were organized since these groups, unlike pharmacists and GPs, contained both RNs and carers.

Focus groups with GPs were conducted in two metropolitan locations in Perth, namely Fremantle (main port south of Perth) and Osborne Park (northern suburb of Perth), Western Australia. These different areas in the metropolitan area were chosen to ensure a wide representation of GPs. Pharmacist participants came from different metropolitan areas and their focus groups were conducted in facilities of the Curtin University, School of Pharmacy. To avoid bias and ensure a wider representation of pharmacists each focus group had pharmacists who were not working in the same pharmacy. Focus groups with RNs/carers were conducted in respective RACF facilities located in different Perth metropolitan areas i.e. Bicton, Myaree and Belmont. RNs/carers were experienced in working at different RACFs. To further ensure a wider representation of participants, RACFs that were managed by different companies were chosen.

GP and pharmacist participants were contacted via telephone to seek agreement to participate and received an information letter and invitation to attend the focus group. GP participants were recruited through contacting their respective Divisions of General Practice aged care panels. Contacting pharmacists that worked with RACFs was a difficult task because there was no available official list of pharmacies that worked with RACFs. These pharmacies were identified by contacting pharmacies initially known to researchers to provide services to RACFs which then provided further information about other pharmacies that serviced RACFs. This was done until a sufficient number of pharmacists agreed to participate in the focus groups. A total of 20 GPs and 14 pharmacies working with RACFs were contacted. Participants for the RNs/carers focus groups were recruited by the RACF manager. The manager who invited RNs and carers to participate was informed about the approximate preferred number of participants in focus group meetings. All focus group participants received an information letter and invitation to attend the focus group and signed a consent form to participate.

A literature review aided the design of focus group questions and protocol [1-3,10-13]. A consultation meeting of researchers and the facilitator of the focus group also assisted in the review and finalization of this process. The final focus groups questionnaire consisted of an opening question (icebreaker), six transition questions and three key questions. The opening question was related to participants’ opinion on current medication supply systems in RACFs. Transition questions pertained to difficulties with current medication supply systems, potential improvements and potential new models of medication supply. Key questions related to model preference and additional training needed.

In order to ensure a degree of neutrality and avoid bias, the focus groups were conducted by a facilitator who was a staff member of School of Pharmacy but not part of the research team. One of the researchers was present at each focus group meeting managing the audio-recording and taking notes about contributions made by each participant. Focus group participants were reimbursed for their time. All focus groups were conducted during February 2009.

Audio-recorded data from the focus group meetings were transcribed into Microsoft Word. In order to perform a secondary content analysis, audio-recorded data were re-listened to and also compared with field notes taken. Transcribed data was imported into NVivo® v8 where it was thematically analyzed by a single independent consultant who discussed and confirmed extracted themes with one of the researchers for consistency. A grounded theory approach was utilized during the process of qualitative analyses. To aid in interpreting the relevance of the comments illustrating a particular theme a ranking system using the symbol † was used. A comment that described a similar issue more than once and in another focus group meeting consisting of same health professionals was marked with a †.

## Results

Out of eight contacted GPs from the Fremantle area four agreed to participate (one GP agreed to participate but cancelled due to an emergency). The focus group with GPs from Osborne Park area had seven participants, out of 12 GPs contacted. Out of 14 pharmacists contacted, a total of 11 agreed to participate in focus group meetings. Pharmacists were divided in two focus groups consisting of five participants (i.e. one cancelled due to an emergency). The focus group with RNs/carers at the Bicton location had six participants. The focus group with RNs/carers at the Myaree and Belmont locations each had five participants.

There were three main themes that emerged in focus groups discussions regarding the medication chart model. These themes pertained to: a) support for its use, b) reviewing the medication chart and c) potential difficulties with using the medication chart as a replacement prescription. Details of these are given below:

a) Support for using the medication chart as a replacement for prescriptions

Using a medication chart model as a replacement for prescriptions to supply medications to RACFs was generally supported by all professions. A RN suggested that medication charts clarify what is given to the resident by instantly knowing the medication the GP has authorized to give. Furthermore, it appeared that in some RACFs, medication charts were already the document by which the nursing staff were guided. The fact that medication charts were already superseding prescriptions was also highlighted by pharmacists. Centralization of medication supply and no variation in frequency of repeat prescriptions when medication charts are used were mentioned as reasons for pharmacists supporting this model. GPs also thought that the medication chart model enabled clarification of medication deliveries from pharmacies to RACFs and centralization of medication supply. According to GPs this model would allow them to maintain control over the type of medications their patients receive. Support statements for using the medication chart as a replacement for prescriptions are illustrated in Table 1.

**Table 1:** Comments illustrating the support for using a medication chart model as a replacement prescription

**Participants**	**Comments to illustrate the theme: support for a medication chart model**
RNs/carer	Most of the time we use the medication chart as an official document. The only time we have a script (i.e. prescription) is if one of our residents has an outside GP, then they come back with the script, we photocopy it and fax it through to the pharmacy and they pick up the script when they deliver the medication
Pharmacists	Facility calls to say the (paper) script written is different to the medication chart. So we go based on the medication profile not the script †
	My experience is that the medication profile always wins over the script. This problem would not be there if the only focus was the medication chart †
GPs	We already have medication charts in most of the facilities and they work well. They are easy to read and understand and I think it is reasonable to understand that if the chart says that the medications are to be delivered we should be able to get away from us having to provide personal (paper) prescriptions †

The use of an electronic version of the medication chart instead of a paper-based one emerged as an option in pharmacists’ focus groups. Some perceived advantages of electronic medication charts included: responding in real time when GPs change residents’ therapy electronically and automatic online claiming. Pharmacists also highlighted the advantage of this model allowing the pharmacist to see what stock would be required to be dispensed ahead of time. Comments of support for using an electronic version for medication charts are illustrated in Table 2.

b) Reviewing the medication chart

**Table 2:** Comments illustrating pharmacists’ support for using the electronic version of the medication chart

**Participants**	**Comments to illustrate the sub-theme: electronic version of the medication chart**
Pharmacists	With an electronic medication profile, the GPs change the medical profile in real time and you respond in real time if he doesn’t change you don’t respond, no change you don’t have to worry about it. The nursing home or hostel becomes central to everything. They retain control of the whole process as they should †
	An electronic medication chart can enable the pharmacist to log in online and dispense the medication whilst the PBS could easily see what had been dispensed and therefore claimed for each month of supply
	With electronic medication profile and no prescriptions you can very accurately see what will be required a month ahead of time to dispense

The need for a regular review of medication charts was emphasized by all health professionals in focus groups. In the RNs and carers’ focus groups, this appeared to be a necessity based on their experience with the current use of medication charts. In order to assist GPs in reviewing the medication chart a pharmacist suggested an expiry date on medication charts so that it makes it necessary for GPs to review, whereas a GP proposed a review interval. This is illustrated in Table 3.

c) Potential difficulties with using the medication chart model

**Table 3:** Comments illustrating the need for regularly reviewing medication charts

**Participants**	**Comments to illustrate the theme: the need for reviewing the medication chart**
RNs/carer	Sometimes I don’t think they review the medication profile because if someone is started on paracetamol as a regular dose, you would still find it as PRN on the bottom so you could find they have had a double dose of paracetamol in a day
Pharmacists	They should be valid for six months as it forces the GP to review the patient regularly
GPs	I foresee that it could work provided we were required to review it on a quarterly basis. I don’t know that I would want have to review it sooner than that, it is just not feasible †

Potential difficulties with using a centralized medication chart as a legal document were identified by all health professionals. Changes to residents’ therapy during weekends were identified by RNs and carers as a current difficulty with using medication charts and therefore urged caution in this regard for any future medication chart model. It was stressed that there may be cases where medication charts do not correspond with what the nursing staff actually administers to the residents as the GP may have not charted the medication. This may occur in cases where GP made the change over the phone and a new list of medications is not supplied by the pharmacy until the next working day. Another potential problem for RNs and carers with using medication charts as central documents was that it may not solve the problem of medications prescribed for short-term or PRN use. This is because the GP in those situations may not be available.

Legible writing with paper-based medication charts, especially when working with duplicates was highlighted by pharmacists as a potential problem. Additionally, claiming medications dispensed also appeared to be a concern with using paper-based medication charts. Potential technical computer problems affecting the process and GPs who may not be comfortable with using the electronic version of medications charts were also highlighted. The main concern GPs had with a prescription-less medication chart model was the potential lack of control over the amount of medications dispensed by the pharmacy. The above potential difficulties are illustrated in Table 4.

**Table 4:** Comments illustrating potential difficulties with using medication charts as sole prescriptions

**Participants**	**Comments to illustrate the theme: potential difficulties with using medication charts as a replacement prescription**
RNs/carer	Medication chart model will not entirely solve our problem. Although the medication chart is your legal document you still need medications prescribed for different conditions at different times like urine infection, vomiting and diarrhoea, constipation so you still need someone to add those medications onto that document
Pharmacists	You could have situations where you are not sure whether that was the original medication chart that had been submitted for claiming
GPs	I would be in favour of using medication charts but that would require somebody to monitor the pharmacies delivering medications. How many prescriptions (i.e. medications) they dispense in our name because we will then no longer be responsible for the number of scripts going out in our name †

## Discussion

This study has explored the use of a centralized medication chart model to replace paper prescriptions for supplying medication to RACFs. The data were collected from the main health professional groups involved in therapeutic management of residents currently living in RACFs. As a result, this study confirmed the use of a prescription-less centralized medication chart model as a potential means to improve the medication supply system to RACFs and identified some issues that would need to be taken into consideration when designing such a model. This study found that there were indications of the medication chart already superseding the existing use of paper prescriptions.

Pharmacists’ support for an electronic version of the medication chart confirms findings of the HMA review who suggested that a paper-based medication chart should be only a transition to an electronic version. The electronic version of the medication chart was supported by stakeholders in the HMA review [5]. An electronic version may address some of the main potential difficulties that were raised by focus group participants in regards to using the medication chart model. These difficulties included: reimbursement of pharmacists, RNs/carers concerns with potential discrepancies between medication charts and medication administration when medications were prescribed during the weekend and GPs concern with the potential lack of control over the amount of medications dispensed by pharmacies (i.e. which the current prescription system maintains). Pharmacists also suggested that this model would allow real-time response to medication orders. The use of this model is likely to be confounded by potential technical problems and push back by some GPs who may feel uncomfortable in using an electronic version of the medication chart model.

Findings from this study could also apply to other countries where medication supply to nursing home residents is dependent upon the supply pharmacy receiving a prescription or medication order in order to continue supplying medicines and hence maintain residents’ therapy. For example, a prescription-less model of supplying medicines would be advantageous in the United Kingdom where currently continuation of supply is dependent on Registered Managers of nursing homes, in many cases, having to give GP surgeries 48 hour notice to produce repeat prescriptions which are then sent to the supply pharmacies [14]. A centralized medication supply system, especially an electronic one where stakeholders have direct access, would make this process unnecessary. Likewise, this system would overcome the potential administrative burden of having to pass written medication orders to the pharmacy by nursing homes’ medical staff (currently the case in the United States of America) therefore enabling more time to be focused on patient care [15].

Overall, the findings of this study suggested that the current use of paper prescriptions in RACFs may unnecessarily burden the supply of medications in this setting. In this regard there were signals that the current use of prescriptions is serving primarily as a means of pharmacists being reimbursed for medications dispensed, as well as GPs retaining control over the amount of medications dispensed by a pharmacy.

The main strength of this study is the representation of GPs, pharmacists as well as RNs and carers in focus group meetings. These health professionals were all experienced in dealing with medication supply to RACFs and all were currently working in at least one RACF. It should be noted that this study was only conducted in Western Australia and did not include participants from other Australian states and territories. This limitation may be considered minimal to the overall study results given the achievement of a saturation point in terms of new ideas and comments made by focus group participants as well as the similarity of medication supply systems to RACFs across the Australian states and territories. In this study no distinction was made between low-care and high-care RACFs as the medication supply system as the need for a prescription to facilitate continued supply is the same between the two categories. Diverse focus groups of health professionals may have facilitated exploration of different perspectives and this represents a potential advantage which may have been missed with the use of homogenous focus groups (i.e. same professionals) [9]. However, homogenous groups of participants in focus group meetings were used with the aim of capitalizing on common experiences of participants [11]. Segmentation of focus group participants also facilitates a comparative data analysis [12]. Additionally, the professional hierarchy of focus group participants (in this study consisting of RNs and carers) may have also affected the data [11]. GPs working in RACFs refer residents to a wide range of specialists including geriatricians and psychogeriatricians. These specialists were not invited to attend focus groups, as in Australia GPs carry the main burden of care for RACF residents.

This study provides insight to policymakers and major stakeholders regarding some of the strengths of using a medication chart model and areas that need addressing when designing such a model. There is a need for further research in terms of the potential economical and clinical impact of substituting the paper prescription system with a medication chart model for supplying medications to RACFs.

## Conclusion

This study has confirmed that stakeholders consider the use of a medication chart model as a potential improvement for supplying medications to RACFs. The current use of paper prescriptions may now be associated more with reimbursement of the pharmacy for medications dispensed and GPs’ retaining control over the amount of medications delivered to RACF residents by pharmacies. An electronic version of a medication chart model appeared to address pharmacists’ concerns related to reimbursement and could allow a real-time response by pharmacists. This system could also address GPs’ concerns regarding the amount of medications dispensed by pharmacy by providing transparency in the supply process.

## Misc

Kreshnik Hoti, Jeffery Hughes and Bruce Sunderland contributed equally to this work

## Competing interests

The authors declare that they have no competing interests.

## Authors’ contributions

KH, JH and BS conceived and designed the study. KH, JH and BS collected the data. KH drafted the manuscript. All authors contributed to interpretation of results and critical review of the final manuscript. All authors critically reviewed the article for important intellectual content. All authors read and approved the final manuscript.

## Pre-publication history

The pre-publication history for this paper can be accessed here:

http://www.biomedcentral.com/1471-2318/12/25/prepub

## References

[B1] The Royal Australian College of General Practitioners Medical care of older persons in residential aged care facilities 2006 4 RACGP, South Melbourne

[B2] Australian Pharmaceutical Advisory Council Guidelines for medication management in residential aged care facilities 2002 3 Commonwealth of Australia, Canberra 7 15

[B3] Aged Care Association Australia (ANHECA) Aged care Australia: the future challenges 2004 ANHECA, Canberra

[B4] Carruthers A Naughton K Mallarkey G Accuracy of packaging of dose administration aids in regional aged care facilities in the Hunter area of New South Wales Med J Aust 2008 188 280 282 18312191 10.5694/j.1326-5377.2008.tb01620.x

[B5] Healthcare Management Advisors The Fourth Community Pharmacy Agreement. Review of the Existing Supply Arrangements of PBS Medicines in Residential Aged Care Facilities and Private Hospitals. Final Report 2009 The Australian Government, Department of Health and Ageing, Canberra online http://www.health.gov.au/internet/main/publishing.nsf/content/pharmacy-4cpa-reviews; (accessed 26/05/2012)

[B6] Bessell T Emmerton L Marriot J Nissen L Improving Australians’ access to prescription medicines: proposed practice models 2005 Pharmacy Guild of Australia, Canberra online http://www.guild.org.au/iwov-resources/documents/The_Guild/PDFs/CPA%20and%20Programs/3CPA%20General/2003-017/2003-017_fr.pdf (accessed 26/05/2012)

[B7] The Pharmacy Guild of Australia The Roadmap – The Strategic Direction for Community Pharmacy 2010, online : www.guild.org.au/roadmap; (accessed 05/02/2012)

[B8] The Australian Government Department of Health and Ageing Supply and PBS claiming from a medication chart in residential aged care facilities. Consultation paper, online http://www.health.gov.au/internet/main/publishing.nsf/Content/fifth-community-pharmacy-agreement-consultations; (accessed 03/02/2012)

[B9] The Australian Government Australian commission on safety and quality in health care. National Residential Aged Care Chart Project, online http://www.safetyandquality.gov.au/our-work/medication-safety/medication-chart/nrmc/; (accessed 28/05/2012)

[B10] Smith F Conducting your pharmacy practice research project 2005 London, Pharmaceutical Press

[B11] Kitzinger J Qualitative research: introducing focus groups BMJ 1995 311 299 302 10.1136/bmj.311.7000.299 7633241 PMC2550365

[B12] Forster D McLachlan H Rayner J The early postnatal period: exploring women's views, expectations and experiences of care using focus groups in Victoria, Australia BMC Pregnancy Childbirth 2008 8 27 10.1186/1471-2393-8-27 18644157 PMC2503951

[B13] Morgan D Focus groups Annu Rev Sociol 1996 22 129 152 10.1146/annurev.soc.22.1.129

[B14] Oakley L Boome H NHS Peterborough Community Services The Management of Medicines in Residential Care Homes Policy and Procedure 2011, online www.peterborough.nhs.uk; (accessed 27/04/2012)

[B15] Washington State Department of Health Tamper Resistant Prescriptions, online http://www.doh.wa.gov/hsqa/trpp/PrescriberFAQs.htm; (accessed 30/04/2012)

